# Abdominal volume computed tomography assessment of body composition in dogs

**DOI:** 10.1186/s12917-018-1768-6

**Published:** 2019-01-08

**Authors:** R. B. S. Turner, G. Hepworth, K. Wilson, D. Tyrrell, F. R. Dunshea, C. S. Mansfield

**Affiliations:** 1 U-Vet Animal Hospital, Faculty of Veterinary and Agricultural Sciences, University of Melbourne, 250 Princes Highway, Werribee, Victoria 3030 Australia; 2 Statistical Consulting Centre, University of Melbourne, 139 Barry Street, Carlton, Melbourne, Victoria 3053 Australia; 3 Faculty of Veterinary and Agricultural Sciences, University of Melbourne, Parkville, Victoria 3052 Australia

**Keywords:** Fat mass, Lean tissue, Bone mineral content, Dual-energy X-ray absorptiometry, DXA

## Abstract

**Background:**

Computed tomography (CT) has been used to estimate body composition and determine tissue distribution in dogs, despite limited validation. This may introduce error into estimates of body composition studies and its effect on health in dogs. Further, the modality has not been validated against dual-energy X-ray absorptiometry (DXA) or over a wide range of dog breeds, ages and sexes. The objective of this study was to validate the use of semi-automated, abdominal volume CT for estimating total body composition of dogs relative to DXA. Twenty-two staff-owned dogs (weighing between 5.1-60 kg) were sedated and underwent full body DXA scan and abdominal CT. Abdominal tissue composition was estimated by CT using semi-automated volume segmentation, over predetermined tissue Hounsfield threshold values. Abdominal tissue composition determined by the various CT threshold ranges was compared to total body composition determined by DXA.

**Results:**

Abdominal tissue composition estimated by CT strongly correlated with the estimates derived from DXA with a small Bland-Altman mean percentage differences in values: total body mass (− 250/2000HU: r^2^ = 0.985; − 1.10%); total fat mass (− 250/-25HU: r^2^ = 0.981; − 1.90%); total lean tissue mass (− 25/150HU: r^2^ = 0.972; 3.47%); and total bone mineral content (150/2000HU: r^2^ = 0.900; − 0.87%). Although averaged CT values compared well to DXA analysis, there was moderate variation in the individual predicted values. There was near perfect inter- and intra-observer agreement in segmentation volumes for abdominal fat.

**Conclusions:**

Abdominal volume computed tomography (CT) accurately and reliably estimates total body composition in dogs, but greater variations may be observed in dogs weighing less than 10 kg.

## Background

Body composition may influence the health of dogs, although this is less studied than in people [1, 2]. A specific focus of veterinary research has been on the effect of obesity on the health of dogs [1, 3]. There is also interest in the influence of other components of body composition such as lean tissue and bone mineral content on health outcomes in dogs [1, 2, 4–6].

Due to this interest, multiple non-invasive methodologies have been developed to assess body composition in dogs [1]. Dual-energy X-ray absorptiometry (DXA) is considered the most reliable and accurate non-invasive means of determining body composition and is commonly used as the “gold standard” to validate other methodologies [1, 7–11]. However, the use of DXA in research and clinical use is limited by lack of access and cost of equipment, the requirement for heavy sedation, the length of the acquisition time, the reduced accuracy incurred by superimposition of tissues, the hydration status, and the limited ability to determine the distribution of tissue within the body [1, 7].

The use of computed tomography (CT) to assess body fat content and distribution has been investigated to some degree in dogs, and in humans is a method of choice for validating other techniques of determining body composition [3, 12–15]. Computed tomography offers several advantages over DXA analysis due to rapid acquisition time, improved spatial resolution, contrast resolution, ability to view structures in three dimensions, and ability to assign quantitative value to tissues of different attenuation (Hounsfield units (HU)). Crucially, CT offers the ability to determine body composition, determine the distribution of tissue within the body, as well as lending itself to automated software analyses [1, 12–14]. The ability of CT to discern tissue distribution in a 3D spatial construct allows more specific research into the association of compartmental fat distribution (such as visceral fat) on the health of dogs, opposed to DXA that can only determine tissue composition in a single plane of tissue [3, 16]. Further, CT is more available in veterinary practice and research institutes, making it a more accessible modality for both clinical and research investigation. Finally, stored CT acquisition data may be analysed and reconstructed retrospectively, to determine body composition of dogs, offering retrospective research opportunities.

Despite this, there is minimal evidence to support the use of CT in determining body composition or tissue distribution in dogs. Ishioka et al established a strong correlation between CT fat area measured at the third lumbar vertebrae, and the body fat content measured by deuterium oxide dilution method in 7 male Beagles (*r* = 0.98) [12]. However, to the authors’ knowledge, the use of CT volume segmentation and CT threshold values to determine body composition and tissue distribution over a wide range of dog breeds, ages and sex has not been validated relative to DXA. Further, the use of fat threshold values of − 135/-105HU for dogs differs from those commonly used in human and feline research [17, 18].

The study aimed to validate the use of abdominal volume CT in estimating total body composition of dogs relative to DXA.

## Results

### Descriptive statistics

The twenty-two dogs in the study consisted of 8 neutered females, 3 entire females, 10 neutered males and 1 entire male. The mean age of the dogs was 4.3 years (interval of 6 months to 9 years). The mean weight of the dogs was 23.4 kg (interval of 5.1-60 kg) with a median body condition score of 6 out of 9 (interval of 4–7).

### Total DXA mass

The total volume of abdominal tissue measured by all threshold value combinations on CT showed near perfect correlation to total DXA body mass (− 250/2000HU: r^2^ = 0.985) (see Table 1). Thresholds with upper values exceeding 2000HU did not increase the total volume estimated, while a maximum threshold of 1000HU was not inclusive of all cortical bone on manual inspection and was excluded from further analyses.

**Table 1:** Linear regression statistics of CT threshold values used to estimate DXA body composition in dogs and corresponding validation of the values predicted by CT abdominal using Lin’s concordance (**r**_***c***_^***2***^), Bland-Altman percentage bias and limits of agreement

Hounsfield Threshold Values To Estimate Body Composition (HU)	Regression Equation	r^2^	Gradient [95% CI]	y-intercept [95% CI]	r_c_ [95% CI]	Percentage Difference in Mass [Limits of Agreement] (%)
Total DXA Mass						
−250/1500	y = 3.41x + 0.09	0.985	3.21 to 3.61	−1.39 to 1.58	0.992 [0.982 to 0.997]	−1.25 [− 14.73 to 12.24]
−250/2000	y = 3.46x + 0.02	0.985	3.26 to 3.65	−1.44 to 1.49	0.993 [0.982 to 0.997]	−1.10 [− 14.17 to 11.98]
− 190/2000	y = 3.49x + 0.01	0.985	3.29 to 3.68	−1.46 to 1.47	0.993 [0.983 to 0.997]	−1.05 [− 14.02 to 11.93]
−190/1000	Excluded^a^					
− 250/3000	Excluded^b^					
Total DXA Fat Mass						
−250/−5	y = 1.58x + 1.29	0.985	1.49 to 1.67	0.95 to 1.61	0.992 [0.982 to 0.997]	−1.53 [−22.41 to 19.35]
−250/− 25	y = 1.70x + 1.35	0.981	1.59 to 1.81	0.99 to 1.72	0.991 [0.978 to 0.996]	−1.90 [−25.63 to 21.82]
−190/− 30	y = 1.76x + 1.39	0.981	1.65 to 1.87	1.02 to 1.77	0.990 [0.977 to 0.996]	−1.88 [−26.09 to 22.33]
− 135/− 105	y = 5.31x + 1.46	0.948	4.73 to 5.89	0.86 to 2.07	0.974 [0.938 to 0.989]	− 1.35 [−40.28 to 37.58]
Total DXA Lean Mass						
0/100	y = 6.30x - 3.81	0.956	5.67 to 6.93	−5.98 to −1.64	0.977 [0.947 to 0.990]	6.06 [−39.28 to 51.41]
−5/150	y = 5.75x - 3.01	0.958	5.19 to 6.31	−5.06 to −0.97	0.978 [0.949 to 0.991]	3.82 [−29.86 to 37.49]
−25/150	y = 5.22x - 2.80	0.972	4.81 to 5.64	−4.44 to −1.16	0.986 [0.966 to 0.994]	3.47 [−26.25 to 33.19]
−29/150	y = 5.14x - 3.04	0.977	4.76 to 5.51	−4.55 to −1.52	0.988 [0.972 to 0.995]	4.87 [−30.43 to 40.17]
−105/150	Excluded^c^					
Total DXA Bone Mineral Content						
152/1000	Excluded^a^					
150/1500	y = 2.58x + 0.08	0.905	2.19 to 2.97	−0.04 to 0.21	0.950 [0.886 to 0.979]	−0.91 [−26.82 to 25.00]
150/2000	y = 2.64x + 0.08	0.900	2.23 to 3.05	−0.05 to 0.21	0.947 [0.879 to 0.977]	−0.87 [−26.86 to 25.12]
150/3000	Excluded^d^					

y is the total body tissue mass in kg estimated by the abdominal CT volume

x is the volume in litres calculated by the abdominal CT using the supplied attenuation threshold values

^a^ Further analyses excluded as manual inspection revealed that cortical bone was excluded from the threshold range

^b^ Further analyses excluded as values did not differ to − 250/2000HU threshold

^c^ Further analyses excluded as manual inspection revealed a large volume of fat was included within the threshold range

^d^ Further analyses excluded as the volumes calculated were exactly the same as the 150/2000HU threshold range

**Fig. 1:**
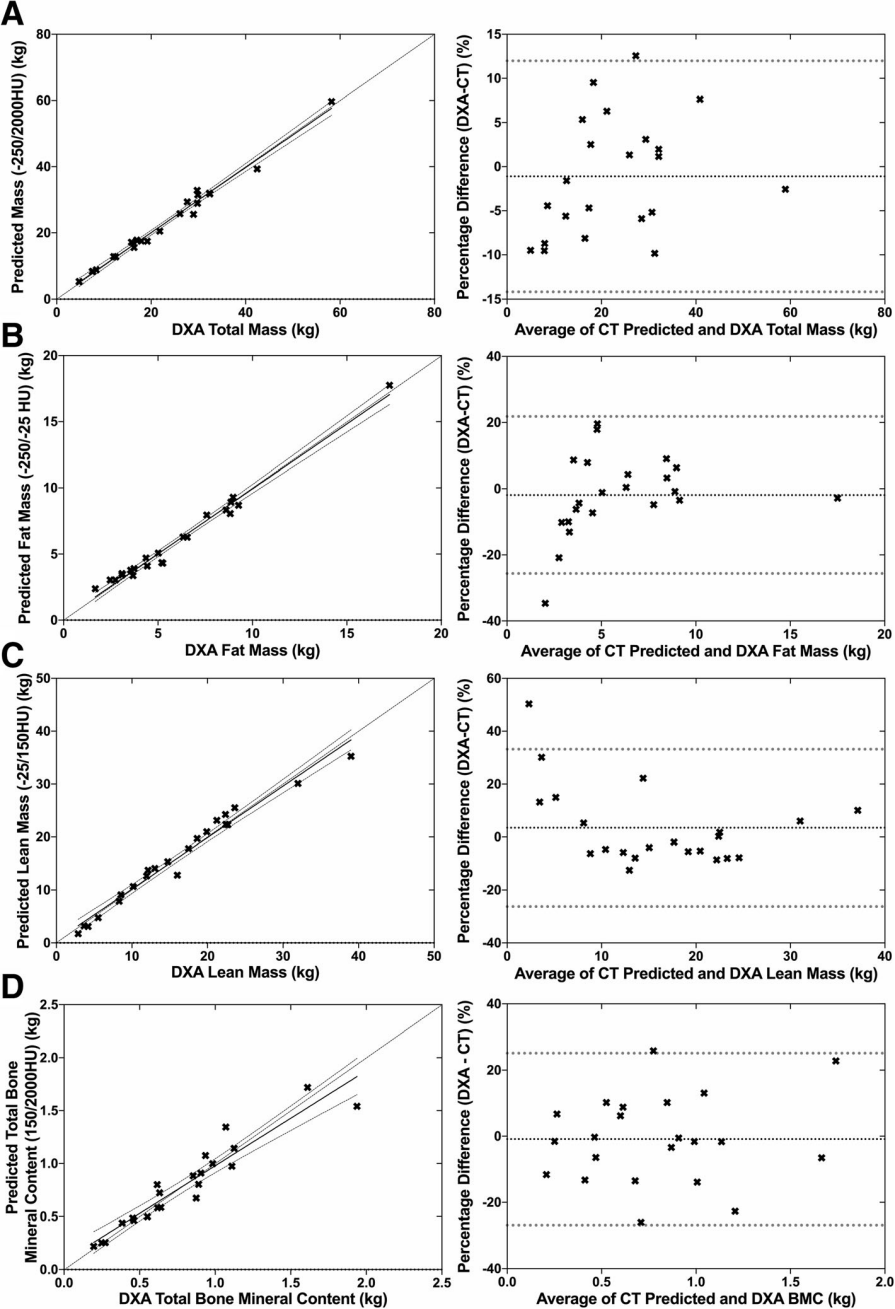
Observed body composition values measured by DXA relative to the values predicted by thresholded volumetric CT by linear eqs. **a**- Total Body Mass. **b** – Total Body Fat Mass. **c**- Total Lean Mass. **d** – Total Bone Mineral Content

Using the regression equations, there was substantial agreement between the mass predicted by CT abdominal volume and the DXA total body mass (− 250/2000HU: r_c_ lower 95% confidence limit (CL) = 0.982). The prediction equations slightly underestimated the DXA body mass (B-A mean percentage difference of − 1.10 and 95% limits of agreement (LOA) [− 14.17, 11.98%]) (see Table 1 and Fig. 1).

### Total fat mass

The total volume of abdominal fat measured by all threshold range combinations on CT showed very strong correlation to total body fat (− 250/-25HU r^2^ = 0.981). Using the regression equations, there was substantial agreement between the total fat mass measured by DXA and the total fat mass predicted by the CT abdominal fat volume (− 250/-25HU r_c_ lower 95% CL = 0.978). The prediction equations slightly overestimated the DXA fat mass (B-A mean of percentage difference of − 1.90 and 95% LOA [− 25.63, 21.82%]) (see Table 1 and Fig. 1).

Fat percentage measured by DXA had a moderate positive correlation with age (r^2^ = 0.284, *P* = 0.011) and no statistically significant relationship with weight, BCS, and sex of the dog. Abdominal fat percentage using absolute volumes had a strong correlation, but poor agreement with total DXA fat percentage (− 250/-25HU divided by − 250/2000HU: r^2^ = 0.563, r_c_ lower 95% CL = 0.154). Total predicted fat percentage had a strong correlation and poor agreement with total DXA fat percentage (Predicted Fat %: r^2^ = 0.778, r_c_ lower 95% CL = 0.737, B-A mean percentage difference of − 0.11 and 95% LOA [− 7.20, 6.97%]).

### Total lean tissue mass

The abdominal lean tissue volume measured by all threshold range combinations on CT showed a strong correlation to total lean tissue mass measured by DXA (− 25/150HU r^2^ = 0.972). Using the regression equations, there was substantial agreement between the total lean tissue mass measured by DXA and the total lean tissue mass predicted by the CT lean tissue volume (− 25/150HU r_c_ lower 95% CL = 0.966). The predictions equation slightly underestimated the DXA lean tissue mass (B-A mean of percentage difference of − 3.47 and 95% LOA [− 26.25 to 33.19]) (see Table 1 and Fig. 1).

Lean tissue percentage measured by DXA had a moderate negative correlation with age (r^2^ = − 0.277, *P* = 0.012) and no statistically significant relationship with weight, and sex of the dog. Abdominal lean tissue percentage using absolute volumes had a moderate correlation, and poor agreement with total DXA lean tissue percentage (− 25/150HU divided by − 250/2000HU: r^2^ = 0.460, r_c_ lower 95% CL = 0.102). Total predicted lean tissue percentage had a very strong correlation and poor agreement with total DXA lean percentage (Predicted lean %: r^2^ = 0.814, r_c_ lower 95% CL = 0.549, B-A mean percentage difference of 1.22 and 95% LOA [− 19.47, 21.9%]).

### Total bone mineral content

The abdominal bone mineral volume (BMV) measured by all threshold range combinations on CT showed a strong correlation to total bone mineral content (BMC) measured by DXA (150/2000HU r^2^ = 0.900). Using the regression equations, there was poor agreement with a moderate negative drift between the total bone mineral content measured by DXA and the total bone mineral content predicted by the CT bone mineral volume (150/2000HU r_c_ lower 95% CL = 0.879). The prediction equations slightly overestimated the DXA BMC (B-A mean of percentage difference of 0.87 and 95% LOA [− 26.86 to 25.12%]) (see Table 1 and Fig. 1).

No significant statistical relationship was found between BMC percentage and DXA with age, weight, and sex of the dog. Abdominal BMC percentage using absolute abdominal volumes had a poor correlation, and poor agreement with total DXA BMC percentage (150/2000HU divided by − 250/2000HU: r^2^ = 0.210, r_c_ lower 95% CI = 0.025). Total predicted BMC percentage had a moderate correlation and poor agreement with total DXA BMC percentage (Predicted BMC %: r^2^ = 0.424, r_c_ lower 95% CL = 0.293, B-A mean percentage difference of − 0.03 and 95% LOA [− 1.13, 1.06%]).

### Reliability of volume segmentation of Total abdominal fat

There was near perfect intra-observer agreement in the segmentation of total abdominal fat (− 250/-25HU: r_c_ lower 95% CL = 0.998, B-A 95% LOA [− 0.44,2.88%]). There was near perfect inter-observer agreement in segmentation of total abdominal fat (− 250/-25HU: r_c_ lower 95% CI = 0.998, B-A 95% LOA [− 1.54, 3.39%]).

## Discussion

Our study showed that total body mass, fat mass and lean tissue mass can be accurately and reliably estimated using abdominal volume computed tomography. However, there was moderate variation in the individual predicted values. The data from this study complements a previous study validating abdominal CT estimation of body fat content relative to the deuterium oxide dilution method [12]. To the authors’ knowledge, our study is the first to validate the use of entire abdominal volume CT against DXA, in determining total body composition in a large sample of mixed breed dogs.

In our study, DXA slightly under-estimated scale weight (bias 2.97%). This has been noted in previous studies (~ 3.4%) and is marginally increased with positioning the dogs in lateral recumbency [7, 19]. Due to the use of staff-owned dogs, lateral recumbency was favoured in our study as there was reduced excitement and movement by the dogs during the acquisition period, induced by the back-and-forth movement of the DXA table. Further, though dorsal recumbency is more precise than lateral recumbency, the use of a standardised position is recommended within a set study [19]. The small negative drift in agreement identified may also be accounted for by the single dog over 50 kg in weight, as the accuracy of DXA has only been validated for dogs between 1.8 and 22.1kgs [7]. The use of an adult human DXA software algorithms instead of a specifically calibrated algorithm in this study likely explains the increased variation in the DXA mass in dogs weighing less than 10 kg, as noted with paediatric patients and other species [20]. Other biases for DXA should also be considered. These include the hydration of the patient, the variable tissue depth, the skeletal maturity of the dogs, the breed conformation, manufacturer’s algorithms used, lack of inter-machine validation, intra-operator variability and the limited data on the accuracy of DXA in dogs [7, 10, 19, 21].

There was an excellent linear isometric relationship between total abdominal volume determined by CT and body weight in this group of dogs. This relationship may prove useful in determining the relationship of abdominal volume to metabolic diseases in future research, and other clinical implication like drug dose determination. However, further investigation is required to establish the strength of this relationship in multiple dog breeds.

In our study, CT consistently under-estimated body weight (1.05–1.25%). This finding has been observed in other species, including dogs, pigs and sheep [13]. The reason for the weight under-estimation in our study is uncertain, however may reflect variation in the supposed isometric relationship of abdominal volume and body weight.

Total body fat content was more accurately predicted by total abdominal volume than fat area measured at the third thoracic vertebrae with CT (r^2^ = 0.985 compared to 0.960 respectively) [12]. The use of volume analysis reduces the variability encountered with area measurements and may allow for comparison of fat content between different breed conformations rather than relying on a single area location [17, 22]. Additionally, the threshold ranges in this study identified the threshold values for fat correlating to clinical experience; feline and human studies more accurately predicted total fat volume than the previously proposed canine range of − 135/-105HU [12]. This finding was also reflected in another study that validated automated software in measuring body composition, which found fat content was best estimated with a Hounsfield range of − 214/7HU [13]. The original paper by Ischioka used histogram analysis to determine the fat threshold range, and more complicated analyses of threshold ranges was used in a feline paper [12, 18]. Though a strong correlation was identified for − 135/-105HU, it had only moderate agreement in predicting total fat content, as it appeared to exclude fat tissue within the segmented regions.

CT slightly under-estimated body fat in this study, however CT may prove to be more accurate in determining fat content than DXA, as it is more reliant on measurements than prediction and is not affected by thickness and superimposition of tissues [7, 21, 23]. The magnitude of the bias in our sample population increases slightly with increasing fat mass in dogs, but when the single outlier is excluded, the bias magnitude becomes constant and is more easily corrected for.

Fat percentage is a more useful clinical and research measurement than absolute fat content, particularly in dogs, where there is a large variety of body sizes and conformations. Within this sample of dogs, the total fat percentage distributed to the abdomen strongly correlated to, but was slightly higher than the total body fat percentage. This would suggest that the overall contribution of fat to the abdominal trunk exceeds that of the total body, but the predicted fat volume and mass can still be used to estimate total fat percentage (LOA –11.55, 8.79% which reduces to − 6.86, 5.85% if the significant outlier is excluded).

The 9-integer scale for body composition score (BCS) developed by Laflamme was used for this study. There was a poor correlation between BCS and total body fat percentage measured by DXA in this study (*r* = 0.407 *P* = 0.060). The low number of subjects [21] and the narrow range of body condition scores [4–7] within the sample population may account for the lack of correlation between BCS and total body fat, considering the reasonably high variability of the error in this subjective methodology [8, 24].

To the authors’ knowledge, this is the first time lean tissue mass of dogs has been predicted by abdominal CT in dogs. There was excellent accuracy in the predicted values; however, the slight underestimation by CT may be explained by the variation created by DXA as described earlier. Further, the large variations likely reflect the conformation of the breeds. Besides, the muscle mass of the dog may best reflect lean tissue percentage compared to visceral lean tissue mass. The decrease in lean tissue mass and skeletal mass with age has been previously reported [4, 5, 25, 26]. This finding may be useful for the assessment of conditions such as age-related sarcopenia, cachexia, and determining the glomerular filtration rate from endogenous creatinine [27].

The strong correlation between abdominal bone mineral volume and total bone mineral content did not translate into a substantial strength of agreement in the prediction equations. This is not surprising, given the small percentage of bone within the abdomen. The improved correlation of using threshold interval greater than the human upper threshold intervals of 1000HU supports the previous finding that dogs have more dense bone compared to humans [6]. The relationship of decreased bone density with age was not identified in our study [28].

There was excellent intra- and inter-observer agreement in CT volume segmentation of total fat volume. The accuracy of the segmentation to reliably predict body composition was not thoroughly evaluated; however, the substantial agreement between the DXA mass and the mass predicted by CT abdominal volume is an indication of the accuracy of the technique.

An additional observation in our study, not formally recorded, was acquisition time parameters for the two modalities. The DXA acquisition time ranged from approximately 8–15 min, and the table movements for each passing scan would sporadically wake the sedated subjects. This would on occasion require additional sedation and repeat acquisition. For CT, the acquisition time ranged from 20-50s, with no requirement to repeat sedation or acquisition. This observation requires formalised recording, however demonstrates the benefit of CT relative to DXA for patient welfare and research efficiency. The CT segmentation methodology used in our study cannot be used in DXA and also has the ability to be applied to regional segmentation, such as the peritoneal cavity, where visceral fat, relative to the extra-peritoneal compartment of the abdomen (subcutaneous space) can be determined. A final benefit of CT compared to DXA, is the ability to retrospectively analyse acquired data, which allows repeat measurements to be performed by two observers, without requiring additional sedation of the dogs, offering improved animal welfare to this research model.

Additional limitations to our study include low sample numbers, limited breed selection and low variation in body condition scores [22]. Despite this, our study indicates that abdominal volume CT assessment of body composition is robust over a range of dog breeds and sizes. Further, our study may have been improved by comparing whole-body CT against DXA or chemical analysis, to limit the error of prediction values.

## Conclusions

Abdominal volume computed tomography (CT) accurately estimated total body composition determined by DXA for dogs weighing between 5.1 and 60 kg, however greater variations in results were observed in dogs less than 10 kg in weight. Further research into the influence of breed, age and body condition on body composition and tissue distribution is recommended. The authors recommend the following Hounsfield threshold ranges to reliably segment body composition: − 250 to -25HU for fat; − 25 to 150HU for lean tissue; and 150 to 2000HU for bone. Our study should provide a methodology for future research into the influence of body composition and tissue distribution on the health of dogs.

## Methods

### Ethics

The University of Melbourne Faculty of Veterinary and Agricultural Sciences Animal Ethics Committee granted ethical approval (Ethics ID: 1613993).

### Animals

Twenty-two dogs of variable breeds were sourced from the staff and students of the U-Vet Animal Hospital, Werribee. All dogs underwent a full physical examination; and were excluded if systemically unwell, or there was a risk associated with sedation. Dogs were fasted for 12 h, weighed and assigned a body condition score out of 9 as described previously [8] by one investigator (RT) on the morning of the imaging analysis. The same scales were used to weigh each dog and were tared daily.

### Experimental protocol

An intravenous catheter was placed in a cephalic vein, and the dogs were sedated by intravenous medetomidine hydrochloride (10 μg/kg of body weight) and butorphanol (100 μg/kg of body weight). The CT and DXA studies were performed sequentially. The dogs were recovered and discharged after the imaging studies.

### Body composition estimation by dual-energy X-ray absorptiometry (DXA)

Body composition was estimated using DXA by the method described previously [7]. A Hologic Discovery W dual-energy X-ray absorptiometer (Hologic, Waltham, MA, USA) was used with switching peak energies of 140/100 kV. Quality assurance and calibration were performed daily using the manufacturer’s anthropomorphic spine phantom and quality control software. The repeatability of DXA measurements in dogs has been reported [29]. Briefly, dogs were placed in lateral recumbency, and the scan field collimated to the size of the dog. Whole body analysis was performed using proprietary purpose-designed computer software and calibrated to body mass. The following variables were estimated: total surface area (cm^2^), bone mineral content (BMC)(g), bone mineral density (g/cm^2^), fat (g), lean tissue mass (g), total body weight (g) and the total body fat percentage.

### Computed tomography (CT)

Volume acquisition was performed with the dog in dorsal recumbency, from the mid-thorax to caudal pelvis, in a transverse plane using a 16-slice CT scanner (Somatom Emotion 16, Siemens, Erlangen, Germany). Helical scan acquisition with a detector configuration of 16 × 0.6 mm gave an effective slice thickness of 0.75 mm with a pitch of 0.8. Technical parameters of 110 kV, and a reference mAs of 120 with a dose modulation technique was employed for each patient. The display field of view was dependent on dog size and an image matrix of 512 × 512 was used. Data was reconstructed using a B31s medium smooth kernel (smoothing algorithm) and viewed in an abdomen (soft-tissue) window. CT data was stored in DICOM format and stored on the hospital PACS.

### CT regional definitions and volume calculation

Proprietary software (Somaris/5 Syngo CT 2014A, Seimens AG, Muenchen, Germany) was used for semi-automated volume quantification of body composition and distribution. Volumes of interest (VOIs) were established by drawing transverse regions of interests (ROIs) at the cranial and caudal margins of the defined abdominal regions (described below). The volume of tissue between these regions was automatically calculated by the software using a defined Hounsfield threshold range of values for the tissue of interest (tissue Hounsfield ranges described below). The software highlighted the volume of tissue for inspection and allowed manual adjustments of the automated boundaries to ensure the defined regions were maintained.

### Abdominal boundaries

**Fig. 2:**
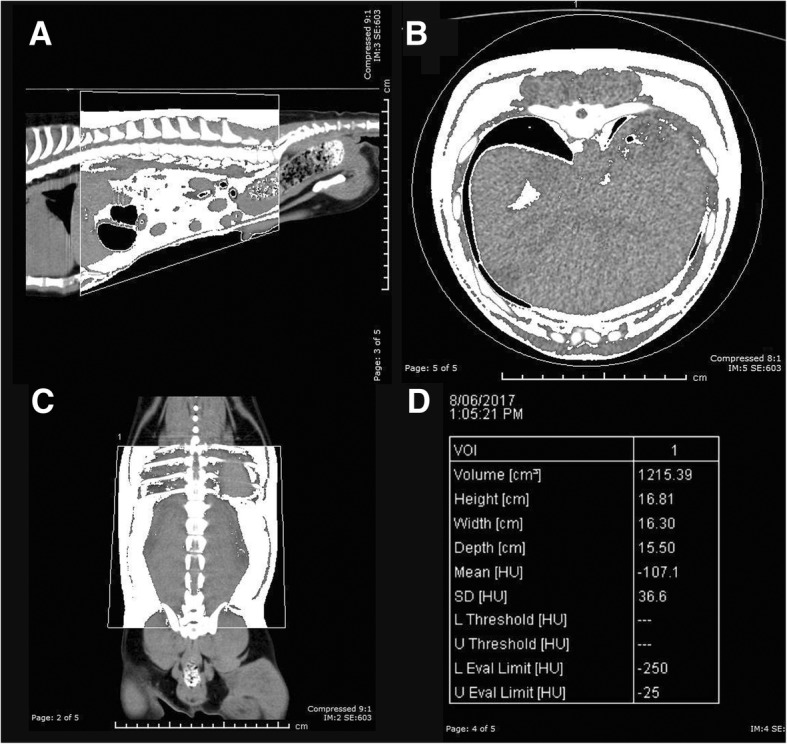
Total Abdominal Fat Segmentation in sagittal (**a**), transverse (**b**), and dorsal (**c**) plane. Data analysis output with threshold values set at − 250/-25HU (**d**)

The volume of the abdomen analysed was defined as all tissue extending between the cranial margin of the tenth thoracic vertebra (T10) to the cranial margin of the first sacral vertebrae (S1). In the case of 8 lumbar vertebrae, the 11th vertebra from S1 was used (T11). This was a fixed region, independent of the diaphragm and bladder positions. Within this region, the total volume of all tissues, the total fat volume, the total lean volume, and the total bone mineral volume (BMV) were calculated (see Fig. 2).

### CT tissue attenuation threshold values

Various Hounsfield threshold values for fat, lean tissue and bone were evaluated using current veterinary and human literature, clinical acumen and subjective assessment of attenuation thresholding as presented in Table 2. The calculated volumes of tissue were compared to the equivalent tissue masses produced using DXA.

**Table 2:** Computed tomographic (CT) threshold values for fat, lean tissue, bone and all abdominal tissue

	Fat (HU)	Lean Tissue (HU)	Bone Mineral Content (HU)	All Tissue (HU)
Final Threshold Values Used in this Study	−250/− 25	−25/150	150/2000	− 250/2000
Threshold Values Trialled	− 250/− 25	−25/150 −5/150 −105/150	150/2000 150/1500 150/3000	− 250/2000 −250/1500 −250/3000 −190/1000 −190/2000
Threshold Values from Literature Used for Comparison				
Dogs [12]	−135/− 105			
People [14, 15, 17, 35]	−190/− 30	−29/150	152/1000	
Cats [18]	−250/−5	−5/150		

### Reliability of volume segmentation of Total abdominal fat

The repeatability (intra-observer variation) and reproducibility (inter-observer variation) for determining total abdominal fat volume, using the semi-quantitative segmentation technique, was assessed on the original volume acquisition of 5 randomly selected dogs. The primary investigator repeated the segmentation for the intra-observer variation, and a radiographer (KW) performed the inter-observer segmentation analysis. The repeat measurements were performed 10 months after the original data analysis. The inter-observer data analysis was performed blinded to the primary author’s results.

### Statistical methods

The required sample size for establishing the expected correlation was calculated to be 4 to 8 dogs. The sample size was computed with G*Power 3.1 [30] using a significance level of 0.05, a power of 0.8, and an expected correlation of 0.8–0.98 [12]. However, as experimental method validation was also being assessed, a minimum of 20 samples, with a rolling sample size to 40 was sought [31, 32].

Relationships between variables were visualised on scatter plots and assessed using linear correlation or t-tests. The assumption of normality or linearity was evaluated, and found to be adequately satisfied. Two-tailed *P*-values were used, and *P* values less than 0.05 were considered statistically significant. The correlation coefficient for continuous variables was described as perfect (*r* = 1.00) very strong (> 0.90), strong (0.70–0.90), moderate (0.50–0.70), poor (0.30–0.50) and negligible (0.00–0.30) correlation [33]. The accuracy and precision of CT abdominal composition to predict DXA body composition were assessed using the regression equations generated. Method validation and strength-of-agreement of the predicted values relative to the DXA values was performed using Bland-Altman (B-A) limits of agreement (LOA) and Lin’s Concordance Correlation Coefficient (Lin’s Concordance Correlation Coefficient; SPSS Syntax; Garcia-Granero, M.; updated 04/2009, https://gjyp.nl/marta/Lin.sps) [34]. Agreement criteria for Lin’s concordance correlation coefficient for continuous variables were described as perfect (r_c_ = 1.00) near perfect (> 0.99), substantial (0.95–0.99), moderate (0.90–0.95) and poor agreement (< 0.90) [32]. The lower 95% confidence limit (CL) for the calculated concordance correlation coefficient was compared to the agreement criteria [32]. Statistical analysis was performed using GraphPad Prism (Graphpad Prism for Mac OS X, version 7.0c, GraphPad Software, La Jolla, CA, USA, www.graphpad.com), SPSS (IBM SPSS Statistics for Mac, version 25, IBM Corporation, Armonk, NY, USA), and Microsoft Excel (Excel 2011 for Mac, version 14.7.2, Microsoft Corporation, Redmond, WA, USA).

## Abbreviations

B-A: Bland-Altman
BCS: Body condition score
BMC: bone mineral content
BMV: total bone mineral volume
CL: confidence limit
CT: Computed Tomography
CV: Coefficient of variation
DXA: Dual energy x-ray absorptiometry
HU: Hounsfield units
LOA: limits of agreement
ROI: Region of interest
VOI: Volume of interest
